# Incidence of macrosomia in Mexico: National and subnational estimations

**DOI:** 10.1371/journal.pone.0276518

**Published:** 2022-12-02

**Authors:** Fermín Avendaño-Alvarez, Eric Monterrubio-Flores, Isabel Omaña-Guzmán, Miriam López Teros, Sonia Hernández Cordero, Karla Muciño-Sandoval, Alejandra Cantoral, Monica Ancira-Moreno

**Affiliations:** 1 Maestría en Nutriología Aplicada, Universidad Iberoamericana, Ciudad de México, México; 2 Observatorio Materno Infantil (OMI), Universidad Iberoamericana, Ciudad de México, México; 3 Centro de Investigación en Nutrición y Salud, Instituto Nacional de Salud Pública, Cuernavaca, México; 4 Departamento de Salud, Universidad Iberoamericana, Ciudad de México, México; 5 Instituto de Investigaciones para el Desarrollo con Equidad, EQUIDE, Universidad Iberoamericana, Ciudad de México, México; University of Cambridge, UNITED KINGDOM

## Abstract

Fetal macrosomia (FM) is a condition with adverse consequences for both mother and offspring. The occurrence of this condition has increased worldwide. The objectives of this study were: (1) to estimate the incidence of FM at the national and state levels in Mexico in 2020; (2) to estimate the incidence of FM stratified by maternal and newborn characteristics; (3) to identify the states with the highest risk of FM; (4) to georeference the incidence of FM. Open data from the Birth Information Subsystem were used. Relative risks were estimated by adjusted Poisson regression models. The national incidence of FM was 2.75%. The entity with the lowest incidence was Mexico City (1.28%) and the most affected states were Sonora (6.20%), Baja California Sur (5.44%), and Sinaloa (5.36%), located in the north of the country. The incidence of FM at the national level is below that reported in the international literature. The results of this study can be used for the design and implementation of programs, public policies, and interventions.

## Introduction

There is no global consensus on the cut-off point for diagnosing fetal macrosomia (FM). According to the American College of Obstetricians and Gynecologists (ACOG), FM is defined as a birth weight higher than 4000 g or 4500 g, independently of the gestational age [1]. Whereas, the World Health Organization (WHO) defines it as birth weight ≥4000 g [2].

FM is a condition with adverse consequences for both mother and offspring. In mothers, it is associated with an increased risk of postpartum hemorrhage, prolonged and instrumental delivery, perineal tears, C-sections, prolonged hospitalization, and puerperal infections. Whereas, in newborns, it increases the risk of shoulder dystocia, clavicular fracture, intrauterine hypoxemia, intensive care unit admission, and death [3–6]. In addition, in the offspring, it increases the risk of chronic non-communicable diseases (NCDs) in adult life, such as cardiovascular diseases, type 2 diabetes mellitus, and obesity [1, 7, 8].

Studies carried out in different populations have identified that maternal obesity, pregestational and gestational diabetes mellitus, advanced age, and parity are maternal factors that increase the risk of FM [7, 9–11]. Male newborns are also at higher risk of presenting FM [9].

In the last 30 years, the incidence of FM has increased worldwide and this increase has been different among the countries [12]. The prevalence of FM in high-income countries is between 5% and 20% and its rise has been attributed in some cases to the increase in maternal obesity and pregestational and gestational diabetes [12, 13]. In Queensland, Australia, the prevalence of FM increased from 12.2% in 1988 to 12.8% in 2005 [14]. Similarly, in Beijing, China, an increase from 6.6% in 1996 to 9.5% in 2000 was observed [2]. Another study conducted in Northwest China found that the incidence of FM increased from 6.0% in 1994 to 8.49% in 2000 and subsequently decreased to 7.83% in 2005 [15]. On the other hand, in a retrospective cohort study conducted in the United States, a decrease in the incidence of FM was observed during the period from 1971 to 2017 (from 8.84% to 8.07%) [16].

In some Latin American countries, the prevalence of FM has been estimated. In Brazil [6], from 2001 to 2014, the prevalence was 5.2%; in Colombia [17] in 2011 it was 3.9%; while in Peru [18] it was 5.8% in 2018. At the time of the present analysis, in Mexico, there is only one published estimation at the national level, which corresponds to 2004–2005; the prevalence in this period was 3.8% [13]. The scarce of information on national estimates of FM, as well as the lack of studies that assess the association of FM with various maternal characteristics such as health status and sociodemographic variables, represent important limitations that must be addressed to understand the current situation in the country.

Considering the above, the objectives of this study are: (1) to estimate the incidence of FM at the national and state levels in Mexico in the year 2020; (2) to estimate the incidence of FM stratified by maternal and neonatal characteristics; (3) to identify the states with the highest risk of FM in Mexico; (4) to georeference the incidence of FM according to newborn’s sex.

## Materials and methods

### Study design

The present is a population-based cross-sectional study designed to estimate the incidence of FM in 2020 using the open-access databases from the Birth Information Subsystem (SINAC for its acronym in Spanish). The inclusion criteria in this research were: live births, gestational age at birth >21 gestational weeks, and birth weight ≥500 g. Data on newborns with missing values of sex, gestational age at birth, or birth weight were excluded. FM was considered as a birth weight ≥4000 g.

### Data sources

#### Birth Information Subsystem (SINAC)

The data were obtained from SINAC [19], which is the system that integrates information on births in Mexico. These data are representative at the national and state levels. From these records, neonatal characteristics (birth weight, sex, birth order, and gestational age at birth) and maternal characteristics (age, place of residence, schooling, marital status, occupation, indigenous language, parity, prenatal care, first medical consultation, and information about pregnancy) were obtained.

### Data analysis

Descriptive analyzes were performed to characterize the study population. For categorical variables, proportions were calculated; while for continuous variables, means and standard deviations (SD) were estimated.

#### FM incidence

The incidence of FM was estimated by dividing the total number of newborns with this condition by the number of births that occurred in 2020 at the national and state levels and sorted by sex of the newborns. The risk of FM was estimated at the national level by state and by sex of the newborn using Poisson regression models with robust estimates. These models were adjusted by newborn’s sex, mother’s state of residence, maternal age, marital status, occupation, schooling, if she speaks an indigenous language, number of prenatal consultations, parity, birth order of the neonate, type of pregnancy (single or multiple) and gestational age at birth. The covariables included in the model were chosen according to the scientific evidence. A p-value <0.05 was considered statistically significant.

In addition, the incidence of FM was estimated according to different maternal and newborn characteristics by dividing the number of newborns with FM by the total number of births in each maternal and newborns category.

All statistical analyzes were performed in STATA V 15.0 Software ®.

#### Georeferencing

For the geographical characterization, the INEGI Geostatistical Framework [20] and the FM incidence database were imported into the free Geographic Information Systems software QGIS® to map the incidences.

## Results

During the study period (January to December 2020), 1,747,847 births were registered. We excluded 95,840 births with gestational age at birth <22 weeks or birth weight <500g, and those with missing data on birth weight and newborns sex. Therefore, the final sample was 1,652,007 births.

### FM incidence at the national and state levels

The incidence of FM at the national level was 2.75% and it was higher in male than in female newborns (3.17% y 2.31%, respectively). This trend was also observed at the state level. The highest incidence was observed in the northwestern states of Mexico: Sonora (6.20%), Baja California Sur (5.44%), and Sinaloa (5.36%). In Sonora, the difference in FM incidence between males and females was 1.5 percentage points (pp), in Baja California Sur it was 2.15 pp, while in Sinaloa this difference was 1.29 pp (Table 1).

**Table 1:** National and state incidence of fetal macrosomia, Mexico 2020.

	Both		Male		Female	
	Births (n)	Incidence % (n)	Births (n)	Incidence % (n)	Births (n)	Incidence % (n)
**National**	1,652,007	2.75 (45,472)	851,970	3.17 (26,994)	800,037	2.31 (18,478)
**Maternal state of residence**						
Sonora	36,544	6.20 (2,266)	18,698	6.94 (1,298)	17,846	5.42 (968)
Baja California Sur	10,527	5.43 (572)	5,360	6.49 (348)	5,167	4.34 (224)
Sinaloa	39,972	5.36 (2,142)	20,391	5.99 (1,221)	19,581	4.70 (927)
Baja California	44,274	5.14 (2,276)	22,695	5.98 (1,358)	21,579	4.25 (918)
Nayarit	16,613	5.08 (844)	8,443	5.82 (491)	8,170	4.32 (353)
Colima	8,872	4.19 (372)	4,617	4.66 (215)	4,255	3.69 (157)
Chihuahua	49,255	4.02 (1,982)	25,221	4.61 (1,162)	24,034	3.41 (820)
Tamaulipas	47,040	3.83 (1,803)	24,073	4.40 (1,060)	22,967	3.24 (743)
Coahuila	45,722	3.68 (1,683)	23,312	4.36 (1,016)	22,410	2.98 (667)
Tabasco	35,613	3.65 (1,299)	18,050	4.22 (761)	17,563	3.06 (538)
Durango	27,098	3.54 (958)	13,867	3.87 (536)	13,231	3.19 (422)
Veracruz	93,650	3.31 (3,103)	48,068	3.75 (1,803)	45,582	2.85 (1,300)
Quintana Roo	24,232	3.24 (786)	12,545	3.67 (460)	11,687	2.79 (326)
Campeche	12,809	3.19 (409)	6,597	3.33 (220)	6,212	3.04 (189)
Jalisco	115,072	2.87 (3,308)	59,376	3.43 (2,034)	55,696	2.29 (1,274)
Oaxaca	59,440	2.84 (1,690)	30,748	3.31 (1,019)	28,692	2.34 (671)
Guerrero	47,896	2.82 (1,352)	24,801	3.21 (796)	23,095	2.41 (556)
Nuevo León	78,949	2.82 (2,225)	40,227	3.26 (1,313)	38,722	2.36 (912)
Zacatecas	25,115	2.81 (706)	12,932	3.36 (434)	12,183	2.23 (272)
Chiapas	72,992	2.71 (1,978)	37,705	3.13 (1,180)	35,287	2.26 (798)
Michoacán	74,001	2.70 (1,999)	38,165	3.09 (1,181)	35,836	2.28 (818)
San Luís Potosí	41,353	2.45 (1,014)	21,590	2.75 (593)	19,763	2.13 (421)
Aguascalientes	20,418	2.32 (474)	10,550	2.69 (284)	9,868	1.93 (190)
Guanajuato	92,643	2.29 (2,125)	47,524	2.62 (1,246)	45,119	1.95 (879)
Querétaro	33,470	1.93 (647)	17,344	2.33 (404)	16,126	1.51 (243)
Hidalgo	36,543	1.77 (646)	18,895	2.04 (386)	17,648	1.47 (260)
Morelos	24,462	1.75 (429)	12,751	2.16 (276)	11,711	1.31 (153)
Yucatán	28,023	1.65 (462)	14,529	1.95 (284)	13,494	1.32 (178)
Puebla	102,285	1.63 (1,672)	52,992	1.91 (1,010)	49,293	1.34 (662)
Tlaxcala	19,577	1.49 (292)	10,114	1.69 (171)	9,463	1.28 (121)
State of Mexico	202,347	1.41 (2,844)	105,393	1.65 (1,744)	96,954	1.13 (1,100)
Mexico City	81,939	1.29 (1,053)	42,686	1.53 (655)	39,253	1.01 (398)

The states with the lowest incidence of FM were: Tlaxcala (1.49%), the State of Mexico (1.41%), and Mexico City (1.28%), located in central Mexico.

**Fig 1:**
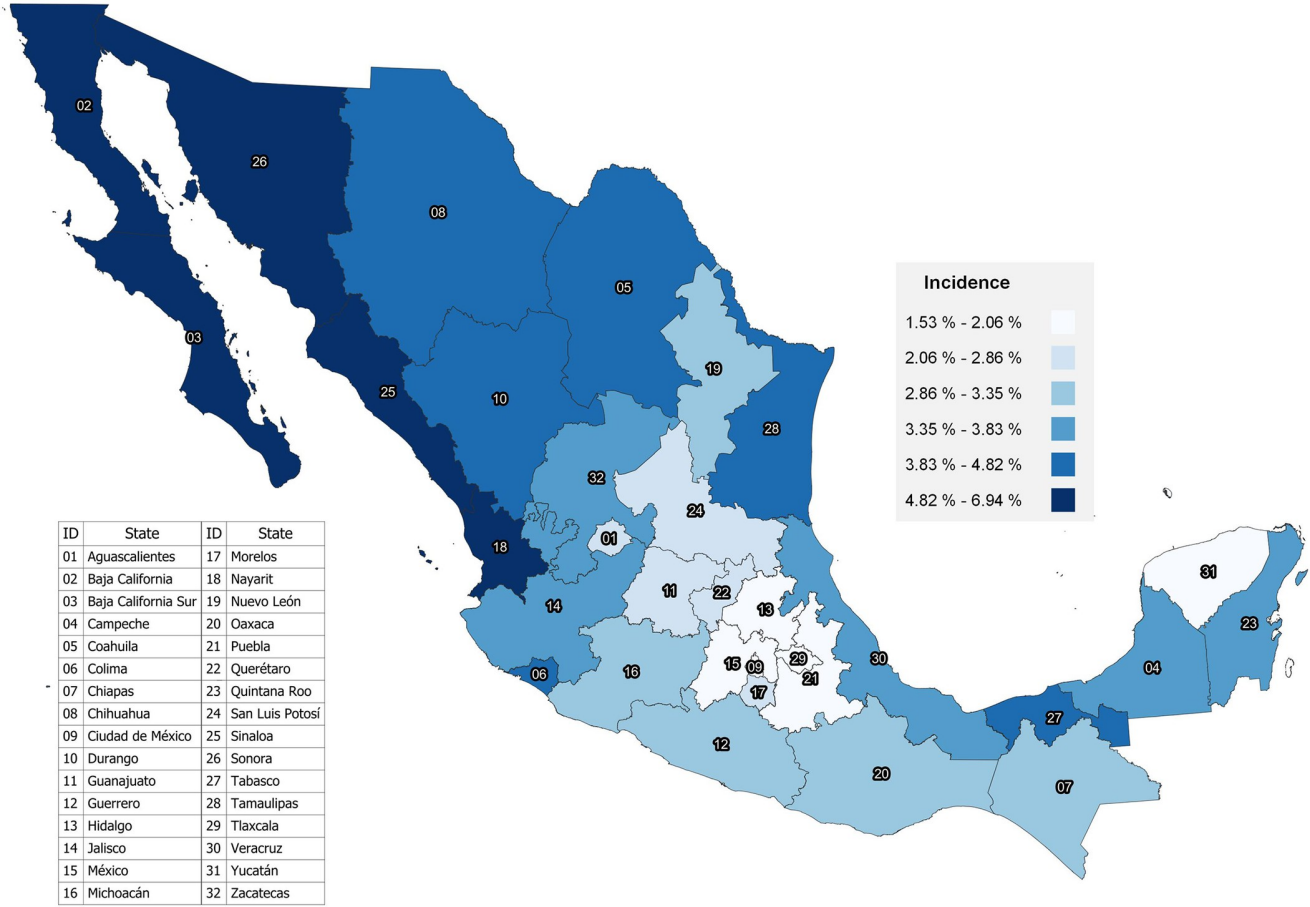
FM incidence in live births in Mexico at the state level in males, 2020.

**Fig 2:**
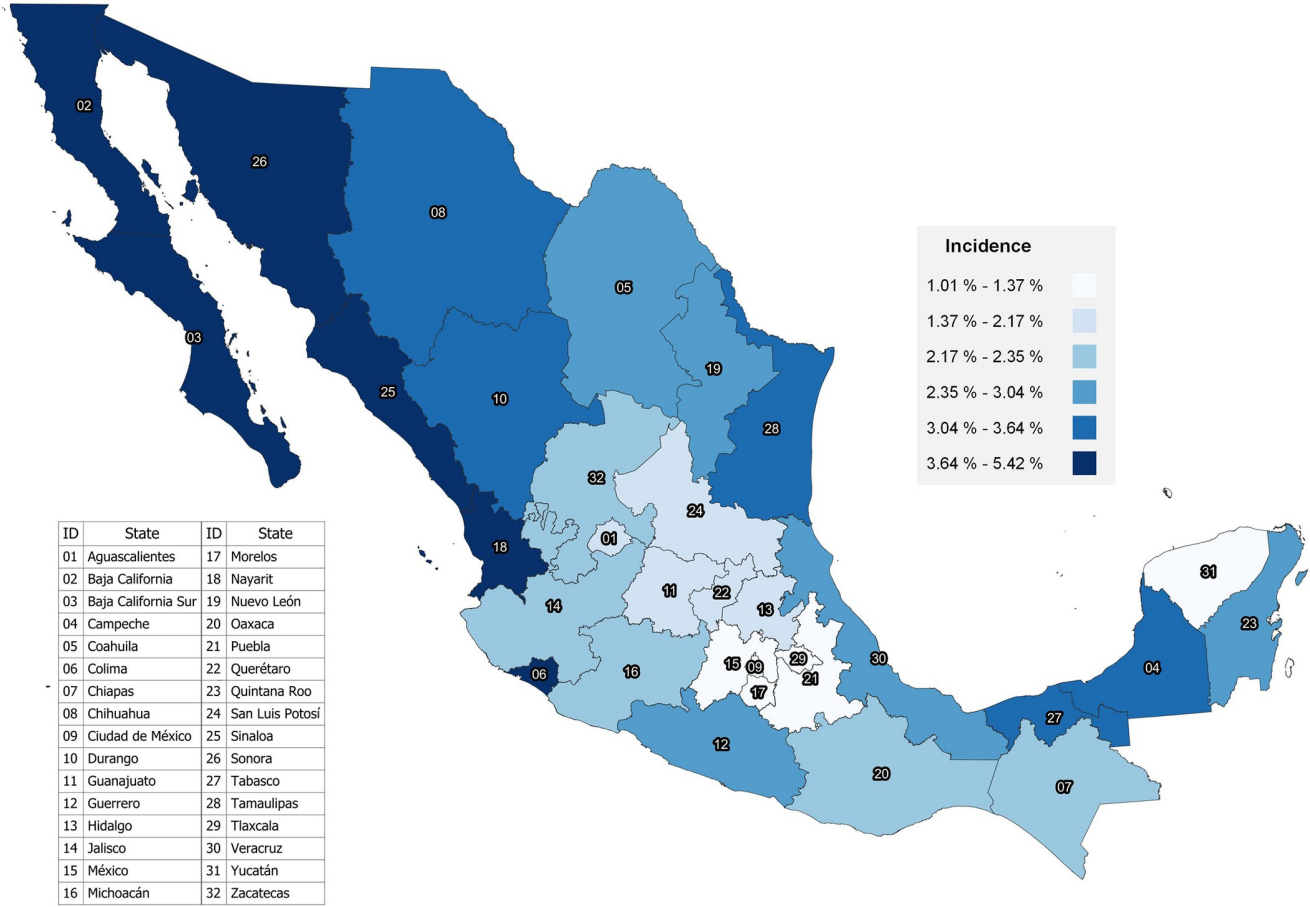
FM incidence in live births in Mexico at the state level in females, 2020.

Figs 1 and 2 represent the maps of FM incidence distribution at the state level by newborn’s sex. The ranges of FM incidence at the state level according to newborn’s sex are very similar.

Regarding the risk of FM by state, Mexico City was considered as the reference category to estimate the ORs since it was the state with the lowest incidence of FM. It was observed that in all the states there was a higher risk compared to Mexico City (Table 2). The states with the highest risk were Sonora (RR = 4.45, 95% CI 4.11–4.82), Sinaloa (RR = 4.08, 95% CI 3.76–4.42), and Baja California Sur (RR = 3.72, 95% CI 3.32–4.18).

**Table 2:** Relative risk (RR) of macrosomia by state, Mexico, 2020.

		Both		Male		Female	
Maternal state of residence	RR (95% IC)		p-value*	RR (95% IC)	p-value*	RR (95% IC)	p-value*
Mexico City	1			1		1	
Sonora	4.45 (4.11–4.82)		<0.0001	4.18 (3.77–4.63)	<0.0001	4.90 (4.31–5.57)	<0.0001
Sinaloa	4.08 (3.76–4.42)		<0.0001	3.81 (3.43–4.23)	<0.0001	4.51 (3.96–5.13)	<0.0001
Baja California Sur	3.72 (3.32–4.18)		<0.0001	3.75 (3.24–4.34)	<0.0001	3.69 (3.06–4.45)	<0.0001
Baja California	3.63 (3.35–3.93)		<0.0001	3.50 (3.16–3.88)	<0.0001	3.83 (3.37–4.36)	<0.0001
Nayarit	3.61 (3.27–3.99)		<0.0001	3.42 (3.01–3.89)	<0.0001	3.92 (3.36–4.58)	<0.0001
Colima	3.10 (2.73–3.53)		<0.0001	2.86 (2.41–3.38)	<0.0001	3.51 (2.86–4.29)	<0.0001
Chihuahua	2.99 (2.75–3.24)		<0.0001	2.86 (2.58–3.18)	<0.0001	3.19 (2.79–3.63)	<0.0001
Tamaulipas	2.72 (2.51–2.95)		<0.0001	2.61 (2.35–2.90)	<0.0001	2.90 (2.54–3.30)	<0.0001
Tabasco	2.68 (2.46–2.92)		<0.0001	2.60 (2.33–2.90)	<0.0001	2.81 (2.45–3.22)	<0.0001
Coahuila de Zaragoza	2.64 (2.43–2.87)		<0.0001	2.63 (2.36–2.93)	<0.0001	2.66 (2.32–3.05)	<0.0001
Durango	2.60 (2.38–2.85)		<0.0001	2.40 (2.13–2.70)	<0.0001	2.92 (2.53–3.37)	<0.0001
Veracruz Ignacio de la Llave	2.43 (2.26–2.62)		<0.0001	2.30 (2.09–2.53)	<0.0001	2.66 (2.35–3.00G)	<0.0001
Quintana Roo	2.27 (2.05–2.51)		<0.0001	2.14 (1.87–2.45)	<0.0001	2.48 (2.10–2.91)	<0.0001
Campeche	2.24 (1.97–2.54)		<0.0001	1.87 (1.58–2.23)	<0.0001	2.83 (2.34–3.41)	<0.0001
Nuevo León	2.13 (1.97–2.31)		<0.0001	2.09 (1.88–2.31)	<0.0001	2.20 (1.93–2.51)	<0.0001
Oaxaca	2.09 (1.92–2.27)		<0.0001	2.03 (1.83–2.26)	<0.0001	2.17 (1.90–2.48)	<0.0001
Jalisco	2.06 (1.91–2.23)		<0.0001	2.06 (1.87–2.26)	<0.0001	2.08 (1.84–2.35)	<0.0001
Guerrero	1.99 (1.82–2.17)		<0.0001	1.87 (1.67–2.10)	<0.0001	2.17 (1.89–2.49)	<0.0001
Zacatecas	1.97 (1.78–2.19)		<0.0001	1.99 (1.74–2.27)	<0.0001	1.94 (1.64–2.30)	<0.0001
Chiapas	1.96 (1.81–2.13)		<0.0001	1.95 (1.76–2.16)	<0.0001	1.99 (1.74–2.27)	<0.0001
Michoacán de Ocampo	1.95 (1.80–2.11)		<0.0001	1.88 (1.70–2.09)	<0.0001	2.06 (1.82–2.34)	<0.0001
San Luís Potosí	1.78 (1.63–1.95)		<0.0001	1.67 (1.48–1.87)	<0.0001	1.97 (1.70–2.27)	<0.0001
Aguascalientes	1.76 (1.56–1.98)		<0.0001	1.72 (1.48–2.00)	<0.0001	1.82 (1.51–2.20)	<0.0001
Guanajuato	1.64 (1.52–1.78)		<0.0001	1.58 (1.43–1.75)	<0.0001	1.75 (1.54–1.99)	<0.0001
Querétaro	1.45 (1.31–1.61)		<0.0001	1.50 (1.32–1.72)	<0.0001	1.37 (1.15–1.63)	<0.0001
Hidalgo	1.35 (1.22–1.50)		<0.0001	1.29 (1.12–1.47)	<0.0001	1.47 (1.24–1.73)	<0.0001
Yucatán	1.23 (1.09–1.38)		<0.0001	1.18 (1.01–1.38)	0.028	1.29 (1.07–1.57)	0.008
Morelos	1.22 (1.08–1.38)		0.001	1.26 (1.08–1.47)	<0.0001	1.16 (0.94–1.42)	0.147
Puebla	1.20 (1.11–1.31)		<0.0001	1.19 (1.07–1.32)	<0.0001	1.23 (1.08–1.40)	0.002
Tlaxcala	1.10 (0.96–1.25)		0.150	1.04 (0.88–1.24)	0.615	1.19 (0.96–1.47)	0.096
State of Mexico	1.03 (0.95–1.11)		0.374	1.01 (0.92–1.11)	0.795	1.07 (0.94–1.21)	0.274

CI, confidence interval

*Significant p value <0.05.

### FM incidence according to maternal and newborn’s characteristics

Table 3 shows the incidence of FM according to maternal sociodemographic characteristics and newborn characteristics. The highest incidence of FM occurred in newborns from mothers in the age group of 35 to 39 years (3.44%), followed by 30 to 34 (3.41%), and older than 40 years (3.05%) (Table 3). Regarding marital status, a higher incidence was observed in newborns from mothers who live apart from their partners (3.28%). In relation to the mother’s occupation, the newborns of artisan mothers had a higher incidence of FM (4.13%), followed by drivers and transport operators (3.57%). On the other hand, the newborns of student mothers and those whose mothers were in the category that included professionals, researchers, professors, and assistants had the lowest incidences (2.16% and 2.32%, respectively). Regarding maternal education, it was observed that newborns whose mothers had no education or elementary school had the highest incidence of FM (2.97%); whereas those whose mothers with postgraduate studies had the lowest incidence (1.73%). On the other hand, a higher incidence of FM was identified in newborns from mothers who did not speak any indigenous language or did not define themselves as indigenous, compared to those who were speakers of an indigenous language or defined themselves as indigenous (2.76% and 2.49, respectively). Of the women who received prenatal care, the highest incidence was in newborns from mothers who received 6 to 9 prenatal visits (2.85%). Regarding parity, the highest incidence was in the group of mothers who had 3 or more previous pregnancies (3.54%).

**Table 3:** Incidence of fetal macrosomia according to maternal and newborn characteristics.

	Both		Male			Female
Maternal and newborn characteristics	Births (n)	Incidence % (n)	Births (n)	Incidence % (n)	Births (n)	Incidence %(n)
Maternal age (years)						
≤19	279,325	1.71 (4,766)	145,696	2.05 (2,980)	133,629	1.34 (1,786)
20–24	461,963	2.45 (11,314)	239,111	2,91 (6,970)	222,852	1.95 (4,344)
25–29	436,198	3.05 (13,313)	224,251	3.49 (7,826)	211,947	2.59 (5,487)
30–34	294,920	3.41 (10,052)	151,285	3.86 (5,834)	143,635	2.94 (4,218)
35–39	143,795	3.44 (4,946)	73,337	3.78 (2,769)	70,458	3.09 (2,177)
≥40	35,267	3.05 (1,075)	18,024	3.40 (612)	17,243	2.69 (463)
**Marital status**						
Single	158,478	2.44 (3,862)	82,409	2.83 (2,331)	76,069	2.01 (1,531)
Widow	2,081	3.08 (64)	1,095	4.02 (44)	986	2.03 (20)
Divorced	2,873	2.82 (81)	1,493	2.55 (38)	1,380	3.12 (43)
Consensual union	908,804	2.66 (24,206)	469,511	3.06 (14,375)	439,293	2.24 (9,831)
Married	535,905	2.96 (15,884)	274,896	3.40 (9,360)	261,009	2.50 (6,524)
Separated	3,958	3.28 (130)	2,000	4.10 (82)	1,958	2.45 (48)
**Occupation**						
Unemployed	32,480	2.65 (860)	16,721	3.19 (533)	15,759	2.08 (327)
Housewives	1,158,841	2.73 (31,658)	597,876	3.14 (18,776)	560,965	2.30 (12,882)
Students	63,393	2.15 (1,366)	32,873	2.56 (843)	30,520	1.71 (523)
Officials in administrative activities	18,712	2.69 (504)	9,752	2.82 (275)	8,960	2.56 (229)
Professionals, researchers, teachers and assistants	84,627	2.31 (1,959)	43,342	2.69 (1,168)	41,285	1.92 (791)
Sales and trade workers	42,503	2.68 (1,138)	21,909	3.04 (667)	20,594	2.29 (471)
Workers in construction, agricultural, livestock and similar activities	19,015	2.55 (484)	9,786	2.81 (275)	9,229	2.26 (209)
Artisans	9,698	4.13 (401)	5,058	4.51 (228)	4,640	3.73 (173)
Transport drivers and operators	26,052	3.57 (931)	13,535	4.03 (546)	12,517	3.08 (385)
Security and surveillance workers	12,129	2.77 (336)	6,215	3.14 (195)	5,914	2.38 (141)
**Education level**						
Elementary school or less	215,750	2.96 (6,394)	111,477	3.32 (3,699)	104,273	2.58 (2,695)
Complete and incomplete secondary /Terminal technician	592,058	2.82 (16,709)	305,582	3.23 (9,876)	286,476	2.39 (6,833)
Complete and incomplete high school/ Terminal technician	509,621	2.76 (14,084)	262,919	3.21 (8,433)	246,702	2.29 (5,651)
Complete and incomplete bachelor’s degree	296,912	2.49 (7,404)	152,427	2.91 (4,429)	144,485	2.06 (2,975)
Complete and incomplete postgraduate	20,570	1.73 (355)	10,653	2.14 (228)	9,917	1.28 (127)
**Considers herself indigenous**						
Yes	117,010	2.50 (2,929)	60,739	2.89 (1,753)	56,271	2.09 (1,176)
No	1,515,871	2.77 (41,932)	781,328	3.19 (24,887)	734,543	2.32 (17,045)
**Speaks an indigenous language**						
Yes	88,629	2.48 (2,201)	46,100	2.85 (1,316)	42,529	2.08 (885)
No	1,541,684	2.76 (42,583)	794,722	3.18 (25,282)	746,962	2.32 (17,301)
**Prenatal care**						
Yes	1,601,320	2.76 (44,177)	825,429	3.18 (26,245)	775,891	2.31 (17,932)
No	43,468	2.52 (1,095)	22,809	2.78 (633)	20,659	2.24 (462)
**Number of prenatal controls**						
0	47,042	2.51 (1,182)	24,671	2.78 (685)	22,371	2.22 (497)
1–3	129,331	2.57 (3,321)	67,270	2.91 (1,959)	62,061	2.19 (1,362)
4–6	492,376	2.75 (13,530)	255,741	3.13 (8,014)	236,635	2.33 (5,516)
6–9	620,773	2.85 (17,700)	319,112	3.29 (10,510)	301,661	2.38 (7,190)
≥10	342,440	2.66 (9,098)	174,773	3.13 (5,470)	167,667	2.16 (3,628)
**Parity**						
1	577,346	1.97 (11,390)	300,598	2.33 (7,013)	276,748	1.58 (4,377)
2	516,031	2.78 (14,326)	266,055	3.23 (8,586)	249,976	2.30 (5,740)
≥3	558,341	3.54 (19,748)	285,180	4.00 (11,393)	273,161	3.06 (8,355)
**Type of pregnancy**						
Single	1,624,133	2.79 (45,339)	837,830	3.21 (26,913)	786,303	2.34 (18,426)
Twin or more	24,493	0.07 (16)	12,434	0.09 (11)	12,059	0.04 (5)
**Gestational age**						
Term (≥37 to ≤42 weeks)	1,526,264	2.92 (44,516)	783,341	3.37 (26,374)	742,923	2.44 (18,142)
Post-term (>42 weeks)	11,426	8.32 (951)	6,694	9.19 (615)	4,732	7.10 (336)

In relation to newborn characteristics, a higher incidence of FM was found when the newborn was a single product in the pregnancy (2.79%), as well as when it was the fifth pregnancy or more (4.48%). The gestational age with the highest incidence of FM was post-term (8.32%).

## Discussion

In 2020, the incidence of FM at the national level was 2.75%, being higher in the north-western states such as Sonora (6.20%), Baja California Sur (5.44%), Sinaloa (5.36%), Baja California (5.15%) and Nayarit (5.08%) where 5 out of 100 births presented macrosomia.

The national incidence was lower than that reported in other studies conducted in Mexico. In a study conducted at the University Hospital of Saltillo [21], an incidence of 18.6% was found in patients admitted between 2012 and 2014. On the other hand, in a General Hospital in Tabasco [22] a prevalence of 5% was observed in 2,100 newborns entitled to the Mexican Institute of Social Security in 2004; while in the Clínica Nova Hospital in Monterrey (n = 1189) [23] it was estimated that the prevalence was of 5.13% in the period from June 2014 to July 2015. It is important to consider that the above-mentioned studies were carried out in different periods of time, with small sample sizes and using records of patients who attended the respective hospitals and who could have greater comorbidity, which limits the comparison with our results. In 2013, a study [13] published the prevalence of FM in 23 countries using data from the WHO Global Survey on Maternal and Perinatal Health. In the same work, a national prevalence of FM of 3.8% was estimated in the period from 2004 to 2005, and this result is closer to that observed in the present study.

The national incidence was also lower than that reported in recent years in the United States (8.07%) [16] and in 2014 in Brazil (5.1%) [6] and Peru (8.1%) [11]. It is important to mention that the lack of FM estimations is a problem that occurs not only in Mexico but also exists worldwide.

Although the national incidence was low, there is high variability between the different states. For example, in the north-western states, the average incidence reached 5%. A possible explanation for this finding is the high rates of obesity in women of reproductive age in this region. According to the National Survey of Health and Nutrition [24] in 2018 the prevalence of obesity in the northern region was 41.6%, whereas in the center it was 33.0%, and in the south 36.1%.

On the other hand, an explanation for the low incidence of FM in the southern states could be maternal height or low birth weight. Maternal height has been identified as a risk factor of birth weight [25] and it has been observed to be lower in the population of the southern states than in the population living in the northern states [26]. Regarding low birth weight, there is a record of a higher incidence in the southeast and Central Mexico; in these regions, a lower incidence of FM was observed [26]. The variability of the incidence of FM in the states of the country could be associated with the different socioeconomic and health conditions of the different regions. Considering the above, it is important to carry out studies in the future to assess the determinants of FM in the different states and regions of the country.

Regarding maternal characteristics, it was observed that the incidence of FM in newborns was higher in the group of women aged 30 to 35 years, in those who had elementary education as the highest level of education, and in those with higher parity. Similarly, other studies have shown that increased maternal age [10], low educational level [17], and parity [15] are risk factors for FM. In addition, maternal age is associated with an increased risk of obesity and gestational diabetes, which in turn increase the risk of FM [7, 10].

This study found that the incidence of FM was higher in male newborns than in females, which is consistent with the literature [3, 6, 8]. Similar results have been reported in Mexico [23], Brazil [6], the United States [16], China [27], and Australia [14]. Male fetuses are known to outgrow female fetuses from early gestation due to a complex interaction between the placenta and fetal sex. It is speculated that this could be due in part to the fact that female fetuses can tolerate an excess of maternal glucocorticoids better than male fetuses; these glucocorticoids play an important role in fetal growth [28]. This mechanism could explain, in part, the differences found between the sexes in different populations. FM is more frequent in newborns who are unique products of pregnancy, as well as in post-term births. According to previous studies [29], there is an increased risk of premature pregnancy or low birth weight in twin pregnancies, but not FM.

It is important to consider the limitations of this study. Although the SINAC database must have a registry of all births that occur in the country, there may be underreporting in the most vulnerable areas of the country (i.e. rural areas). However, births in these places represent a low percentage of total births, so they would not have a significant impact on the estimation of the incidence of FM [26]. Other inherent limitations to the database used in this work is the possible error in the measurement of birth weight or its record, as well as the lack of maternal anthropometric variables (i.e. pre-gestational Body Mass Index) that are associated with birth weight.

These estimations may contribute to the design and implementation of interventions, public policies, and programs aimed at improving maternal and child health and their nutritional status. The regionalization at the state level allows the identification of vulnerable areas that deserve special attention.

In conclusion, this study is an effort in the systematization of statistics in the monitoring of FM in Mexico. It is necessary that future studies estimate the incidence of FM at the municipal level in order to identify the most vulnerable regions affected by this condition in each state. In addition, it would be important to identify the risk factors associated with FM in order to generate evidence that contributes to the design of policies and programs that aim to reduce this condition.
